# The complete mitochondrial genome of Indian cattle (*Bos indicus*)

**DOI:** 10.1080/23802359.2018.1437836

**Published:** 2018-02-10

**Authors:** R. Kumar Pramod, Dinesh Velayutham, Sajesh P. K., Beena P. S., Anil Zachariah, Arun Zachariah, Chandramohan B., Sujith S. S., Sam Santhosh, Sosamma Iype, Ganapathi P., Bangarusamy Dhinoth Kumar, Ravi Gupta, George Thomas

**Affiliations:** a AgriGenome Labs Pvt. Ltd, Kochi, India;; b Vechur Conservation Trust, Thrissur, India;; c Department of Forest and Wildlife, Government of Kerala, India;; d National Institute of Science Education and Research, Bhubaneswar, India;; e SciGenom Research Foundation, Thrissur, India;; f Bargur Cattle Research Station, Tamil Nadu Veterinary Animal Sciences University, Chennai, India;; g Medgenome Labs Pvt. Ltd, Bengaluru, Karnataka, India

**Keywords:** Indian cattle, *bos indicus*, *bos taurus*, mitogenome

## Abstract

India has 40 distinct zebuine cattle breeds with different adaptability and production traits. In the present study, we report the complete mitochondrial genome sequence of Indian cattle for the first time. The mitogenome contains 13 protein-coding genes, 22 transfer RNA genes, two ribosomal RNA genes and a control region (D-loop region). The phylogenetic analysis showed close genetic relationship among the Indian cattle breeds studied, where as, distinct genetic differences were observed between *Bos indicus* and *Bos taurus* cattle. Our results will expand genomic information for further studies on evolution, domestication and conservation of indigenous cattle breeds in India.

India has a large indigenous livestock population includes 40 distinct *Bos indicus* cattle breeds. An extensive and different range of agro-ecological zones in India have assisted in the development of each breed (Sharma et al. 2015). Indian cattle breeds are broadly categorized into dairy, draft and dual purpose breeds based on their utility either in dairying or in agricultural work. However, majority of the draft breeds are under severe neglect resulting in continuous decline of their germplasm. The introduction of highly productive exotic breeds and demographic pressure are also contributing to the loss of precious traits or reduction in population size of indigenous breeds. Therefore, the genetic characterization would be helpful in better understanding of cattle biodiversity as well as formulating action plans on conservation and management of the local breeds.

In this study, we sequenced and characterized the complete mitogenome of six *Bos indicus* cattle breeds and one unrecognized cattle of South India. Blood samples were collected from Tharparkar (Rajasthan, 27.6348° N; 75.1725°E), Kankrej (Gujarat, 23.2700°N; 69.6699°E), Gir (Gujarat, 21.5154°N; 70.4564°E), Ongole (Andhra Pradesh, 15.5057°N; 80.0499°E), Kangayam (Tamil Nadu, 10.3673°N; 77.9803°E) and Vechur (Kerala, 9.5916°N; 76.5222°E) cattle breeds and from an unrecognized cattle population, Kasargod cattle (Kerala, 12.4387°N; 75.2012°E). First, the mtDNA was amplified using two pairs of primers, designed from reported complete mtDNA sequence of *Bos taurus* spp. (GenBank accession no. AF492351). The genome sequence library was constructed by ligating specialized adapters in both ends of sheared amplicon using NEBNext® Ultra™ II DNA Library Prep kit (Illumina, San Diego, CA). The libraries were subjected to sequencing on Illumina HiSeq 2500 platform (Illumina, San Diego, CA). After sequencing, the filtered reads were mapped against a reference sequence, *Bos indicus* (Zwergzebu breed, Genbank accession no. AF492350) complete mitochondrion (Li and Durbin 2009).

The complete mtDNA sequences of cattle breeds studied were deposited in NCBI’s Sequence Read Archive under BioProject ID: PRJNA408207. The mitogenome content of Indian cattle includes 13 protein-coding genes, two ribosomal RNA (12S and 16S rRNA) genes, 22 transfer RNA (tRNA) genes and the control region of 909 bp (*D-loop*) as found in other mammals.

Mitochondrial DNA sequence of studied breeds was compared with published mitochondrial genomes of *Bos indicus* and *Bos taurus* cattle breeds from NCBI database. Maximum likelihood based phylogenetic analysis performed with consensus sequence of each breed (Tamura and Nei 1993). In the phylogenetic tree, *Bos indicus* and *Bos taurus* cattle were present in two separate main clades (Figure 1). The analysis divided *Bos indicus* clade into subclades containing North Indian and South Indian cattle breeds. The South Indian breed, Ongole, was found to be close to North Indian breeds. Interestingly, the reference, European dwarf zebu cattle (Zwergzebu) was placed in the South Indian group.

**Figure 1.**
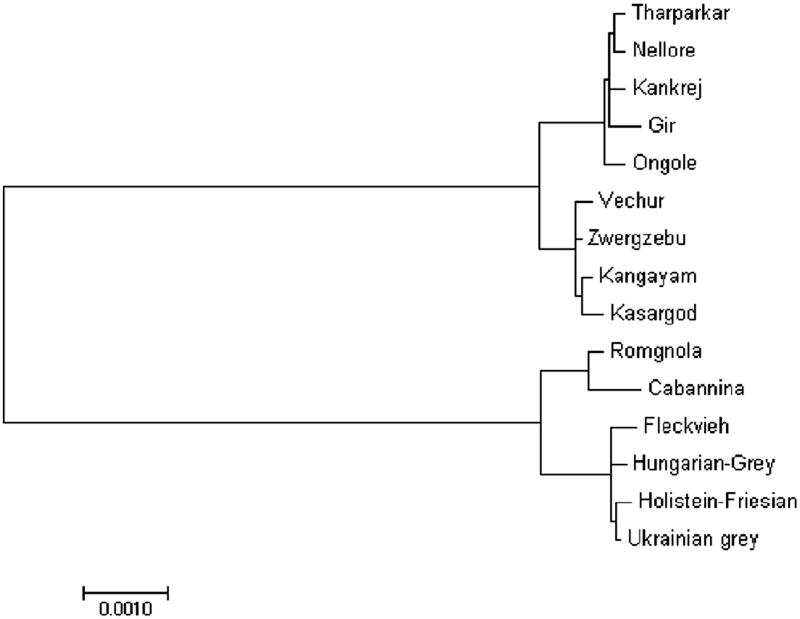
Phylogenetic analysis of cattle, based on the complete mitochondrial DNA sequence. Phylogenetic relationship between mtDNA sequences of Indian cattle and other available cattle mitogenomes were analyzed using maximum-likelihood method. Accession numbers of other breeds used as follows: Zwergzebu AF492350, Nellore NC005971, Holstein-Friesian DQ124418, Ukrainian Grey GQ129208, Hungarian Grey GQ129207, Fleckvieh AF492351, Romgnola FJ971080, and Cabannina EU177867.

In this study, we provided the mitogenome sequence of Indian cattle breeds, in future which may help in prioritization and designing of the conservation plans.
